# A detailed 3D volumetric magnetic resonance arthrographic analysis in a spiral ligament and anteroinferior capsuloligamentous complex of the shoulder joint

**DOI:** 10.1007/s00256-025-04954-x

**Published:** 2025-05-26

**Authors:** Yusuf Yahsi, Hayri Ogul, Rodi Ertogrul, Zakir Sakci, Derya Guclu, Mustafa Ozdemir

**Affiliations:** 1 Department of Orthopedic Surgery, Medical Faculty, Istanbul Medipol University, Istanbul, Turkey; 2 Department of Radiology, Medical Faculty, Istanbul Medipol University, Istanbul, Turkey; 3 Department of Radiology, Health Sciences University, Umraniye Training and Research Hospital, Istanbul, Turkey; 4 Department of Radiology, Medical Faculty, Duzce University, Duzce, Turkey

**Keywords:** Spiral GHL, Middle GHL, AB-IHGL, MR arthrography

## Abstract

**Objectives:**

This study aimed to delineate the anatomical characteristics of the spiral glenohumeral ligament (SpiGHL), middle glenohumeral ligament (MGHL), and anterior band of inferior glenohumeral ligament (AB-IGHL), as well as their potential interrelationships, using MR arthrographic images from a large patient population.

**Methods:**

All patients underwent 3D volumetric magnetic resonance (MR) arthrography sequences in addition to conventional MR imaging. All MR arthrography images were retrospectively assessed by two radiologists with expertise in arthrography. The origins and insertions of SpiGHL, MGHL, and AB-IGHL, along with any variations and interrelationships, were carefully examined and documented. These findings were statistically analyzed according to the patient’s age, gender, and laterality of the shoulder (right or left).

**Results:**

The study included 190 shoulder MR arthrographies, identifying SpiGHL in 15 patients (7.9%). Among the 190 patients, 20 (10.5%) had an AB-IGHL with a high insertion on the anterior glenoid margin. In 10 of 15 patients with SpiGHL (66.6%), a conjugation of SpiGHL and MGHL was observed. MGHL hypoplasia was observed in 11 MR arthrographies. Of the 11 patients, 5 (45.5%) also presented with SpiGHL. The incidence of SpiGHL was significantly higher in the group with hypoplastic MGHL. Additionally, the average age of patients with a high-origin AB-IGHL was significantly lower than that of patients without this feature.

**Conclusions:**

Knowing the anterior capsuloligamentous detailed anatomy of the shoulder joint and its possible variations is important in correctly interpreting the pathologies of this region. The spiral GHL and its possible relationship with other ligaments can be defined in detail on MR arthrography.

## Introduction

The glenohumeral ligaments (GHLs) are integral components of the shoulder joint, providing both static and dynamic stability by reinforcing the joint capsule and limiting excessive motion [1]. These ligaments—classified classically as superior GHL (SGHL), middle GHL (MGHL), and inferior GHL (IGHL)—are composed of dense connective tissue and exhibit distinct anatomical features [2]. The SGHL primarily resists inferior translation of the humeral head, whereas the MGHL limits anterior translation, particularly in mid-range abduction [3]. The IGHL, comprising anterior and posterior bands and the axillary pouch, is the primary stabilizer against anterior and posterior dislocations during abduction and external rotation [4]. Biomechanically, the GHLs work synergistically with the rotator cuff muscles, labrum, and joint capsule to maintain congruency between the humeral head and the glenoid fossa, even under substantial load [4]. Clinical literature has described the variability and more detailed anatomic properties of these structures [5]. Understanding the nuanced roles and pathologies of these ligaments is essential for effectively diagnosing and managing shoulder instability and related disorders.

**Fig. 1:**
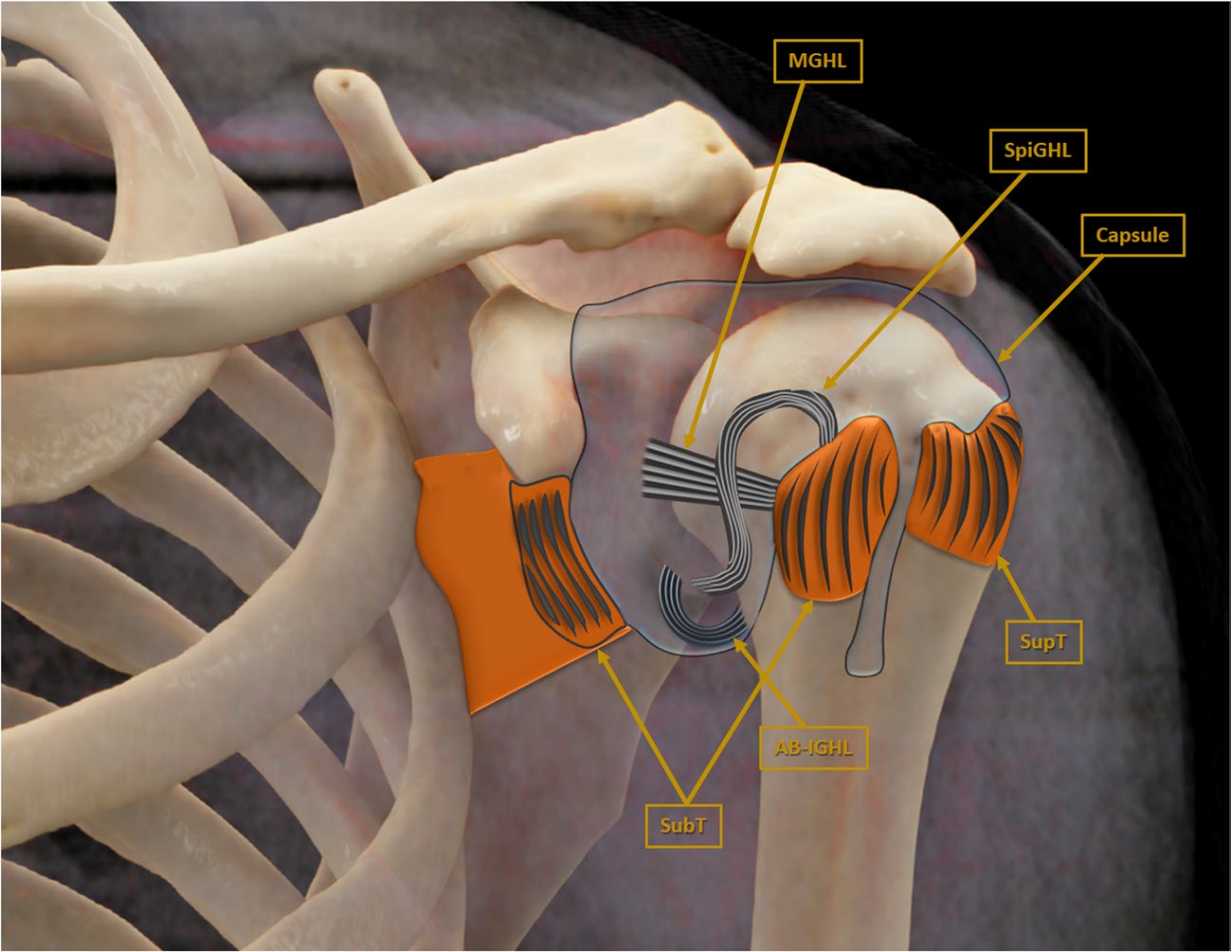
Illustration demonstrating anteroinferior capsuloligamentous structures of the glenohumeral joint in coronal plane. AB-IGHL = anterior band of superior glenohumeral ligament; capsule = glenohumeral joint capsule; MGHL = middle glenohumeral ligament; SpiGHL = spiral glenohumeral ligament; SubT = subscapularis tendon; SupT = supraspinatus tendon

The spiral GHL (SpiGHL), a lesser-known structure of the anterior shoulder capsule, plays a pivotal role in stabilizing the glenohumeral joint, particularly by supporting anterior stability and coordinating with adjacent capsuloligamentous structures [6]. First described as a distinct anatomical entity in detailed cadaveric studies, the SpiGHL is characterized by its oblique course originating from the infraglenoid tubercle and blending with the subscapularis tendon at its insertion on the lesser tubercule (Fig. 1) [6]. Although its biomechanical significance is increasingly recognized, particularly in preventing excessive anterior humeral translation, injuries or pathological alterations to the SpiGHL remain underreported. Damage to this ligament, such as elongation or partial tearing, has been observed in association with anterior instability and repetitive stress, particularly in athletes [7]. However, further studies are necessary to elucidate its precise clinical implications and to standardize diagnostic criteria, as its subtle anatomical presence can challenge identification on imaging and arthroscopic evaluation.

Radiologically, the SpiGHL is a relatively underrecognized and insufficiently characterized anatomical structure. This study aimed to elucidate its existence, morphological attributes, and attachment patterns through detailed magnetic resonance (MR) arthrographic analysis, thereby contributing to a more comprehensive understanding of its anatomical and clinical significance.

## Material and methods

A total of 200 MR arthrographic procedures were performed on 200 shoulders across 198 patients, with two patients undergoing examinations on both shoulders. The primary indications for MR arthrography included Bankart and Bankart variant labral tears, superior labrum from anterior to posterior (SLAP) lesions, rotator cuff injuries, capsuloligamentous damage, and internal–external shoulder impingement syndromes. The MR arthrographic evaluations of the 198 patients were conducted between January 2022 and February 2025. The study was conducted in accordance with the principles of the Declaration of Helsinki. Ethical approval for the study was obtained from our university’s local ethics committee, and signed informed consent forms were obtained from all included patients.

Of 200 shoulder MR arthrographies performed, 10 were excluded from the study for various reasons. MR arthrography images of the remaining 190 shoulders were retrospectively analyzed by two radiologists with 18 and 5 years of experience. The radiological evaluation was performed with the double-blind revision method. In controversial cases, the radiologists reached a final conclusion based on consensus for each case in evaluating the intra-articular course and origin–termination points of the MGHL, SpiGHL, and anterior band of inferior GHL (AB-IGHL). The researchers also assessed the presence, absence, hypoplasia, and tear of these ligaments. Patients with conjugation of MGHL and SpiGHL were noted. As MGHL and AB-IGHL are extensively described in the literature and are commonly reported in routine practice, no difficulties were encountered in this regard. However, when defining SpiGHL, the criteria established by Merila et al. were employed [6]. These encompass the diagonal trajectory from the glenoid origin to the insertion at the lesser tuberosity, a distinct demarcation from the middle glenohumeral ligament (MGHL) and the anterior band of the inferior glenohumeral ligament (AB-IGHL) and a uniform manifestation across at least two orthogonal planes. Because of the absence of histological correlation, capsular folds measuring less than 2 mm were not identified as SpiGHL.

In all MR arthrography procedures, injections were administered as an outpatient process. All these procedures were performed under ultrasound guidance using a posterior approach. A 20-gauge needle facilitated the administration of 12–20 ml injection volume under sterile conditions to ensure adequate joint distension. To optimize the contrast effect, the gadolinium chelate (0.5 mmol/l gadopentetate dimeglumine, Magnevist; Bayer Schering Pharma, Germany) was diluted with saline at a ratio of 1/200 (0.1 ml contrast media diluted by 20 ml normal saline).

MR arthrography procedure was started within 30 min at the latest after the intra-articular injection. All MR arthrographic images were acquired using a Siemens MAGNETOM Skyra 3 T scanner equipped with an 8-channel surface shoulder coil. The MR arthrography imaging protocol included fat-suppressed transverse, sagittal oblique, coronal oblique, and nonfat-suppressed sagittal oblique images with a 3-mm slice thickness. In addition to the conventional MR arthrography images, for each shoulder was also performed three dimensional (3D) volumetric T1-weighted MR arthrography and thin-slice T2-weighted MR arthrography sequences. The MR arthrography sequence parameters are summarized in Table 1. Both conventional and volumetric sequences were assessed on the “syngo.via” workstation by two researchers. Pathologies and variations associated with MGHL, SpiGHL, and AB-IGHL and the attachment levels of SpiGHL and AB-IGHL to the glenoid bone were determined. An AB-IGHL insertion at the 1–3 o’clock position on the glenoid bone is defined as a high-positioned AB-IGHL [8]. Special attention was given to determining whether there was a confluence between SpiGHL and MGHL, and patients exhibiting this confluence were documented.

**Table 1:** 3 T MR scanner routine MR arthrography sequence parameters

Sequence	TR/TE (ms)	ETL	Plane	Flip angle (degrees)	Fat suppression	Slice thickness (mm)	Intersection gap (mm)	Matrix size	FOV (mm)
TSE T1	565/10	8	Oblique sagittal, oblique coronal, and transverse	–	−, +, +	3	3.5	256 × 256	160
TSE T2	4800/70	8	Oblique coronal	–	–	1	1.2	320 × 192	160
3D T1 VIBE	8/3.5	–	Oblique coronal	11	+	0.6	0	512 × 512	160

To evaluate the findings obtained from the study, statistical analyses were performed using IBM SPSS Statistics 22 software (IBM SPSS, Turkey). The suitability of the parameters for normal distribution was assessed using the Shapiro–Wilk test during the evaluation of the study data. Descriptive statistical methods (mean, standard deviation, frequency) were utilized, and Student’s *t*-test was applied for comparisons between two groups for parameters exhibiting a normal distribution when comparing quantitative data. For qualitative data comparisons, Fisher’s exact test, Fisher–Freeman–Halton test, and continuity (Yates) correction were employed. Significant differences were evaluated at the *p* < 0.05 level.

## Results

**Fig. 2:**
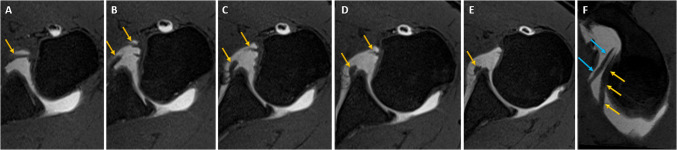
Consecutive axial **A**–**E** and oblique sagittal **F** thin-slice VIBE T1-weighted MR arthrograms reveal high-origin AB-IGHL (yellow arrows) inserting into 2 o’clock quadrant and normal MGHL (blue arrows)

**Fig. 3:**
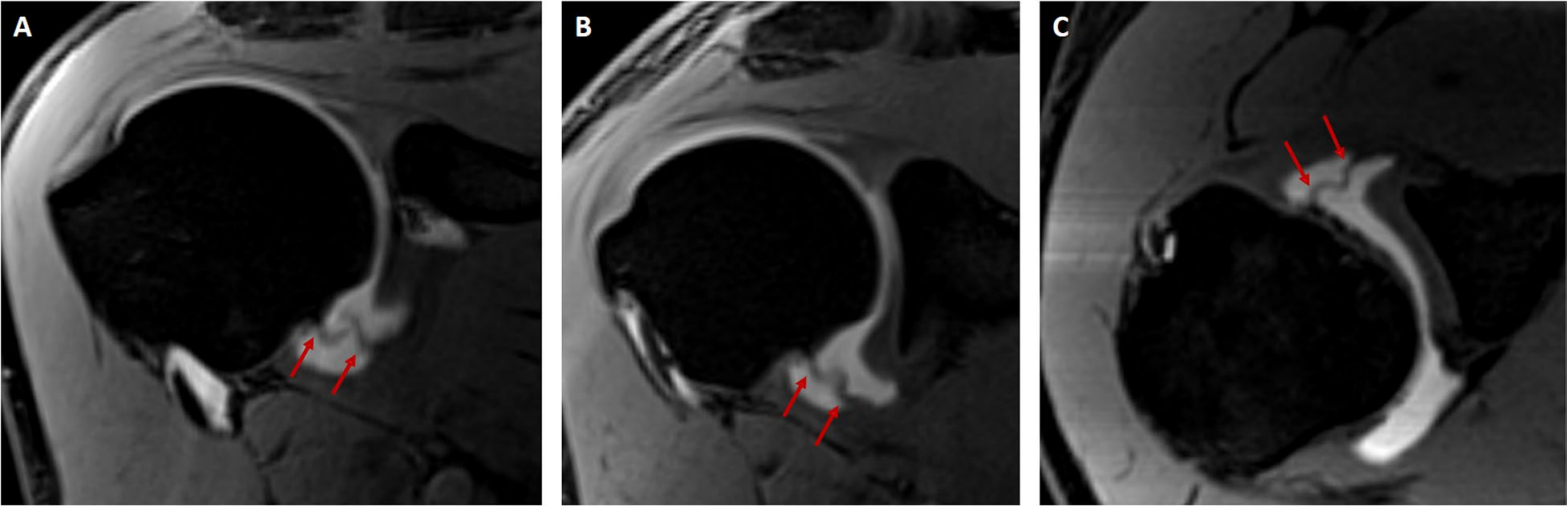
Consecutive oblique coronal **A**, **B** and axial **C** thin-slice VIBE T1-weighted MR arthrograms show SpiGHL (red arrows) without conjugation with MGHL

**Fig. 4:**
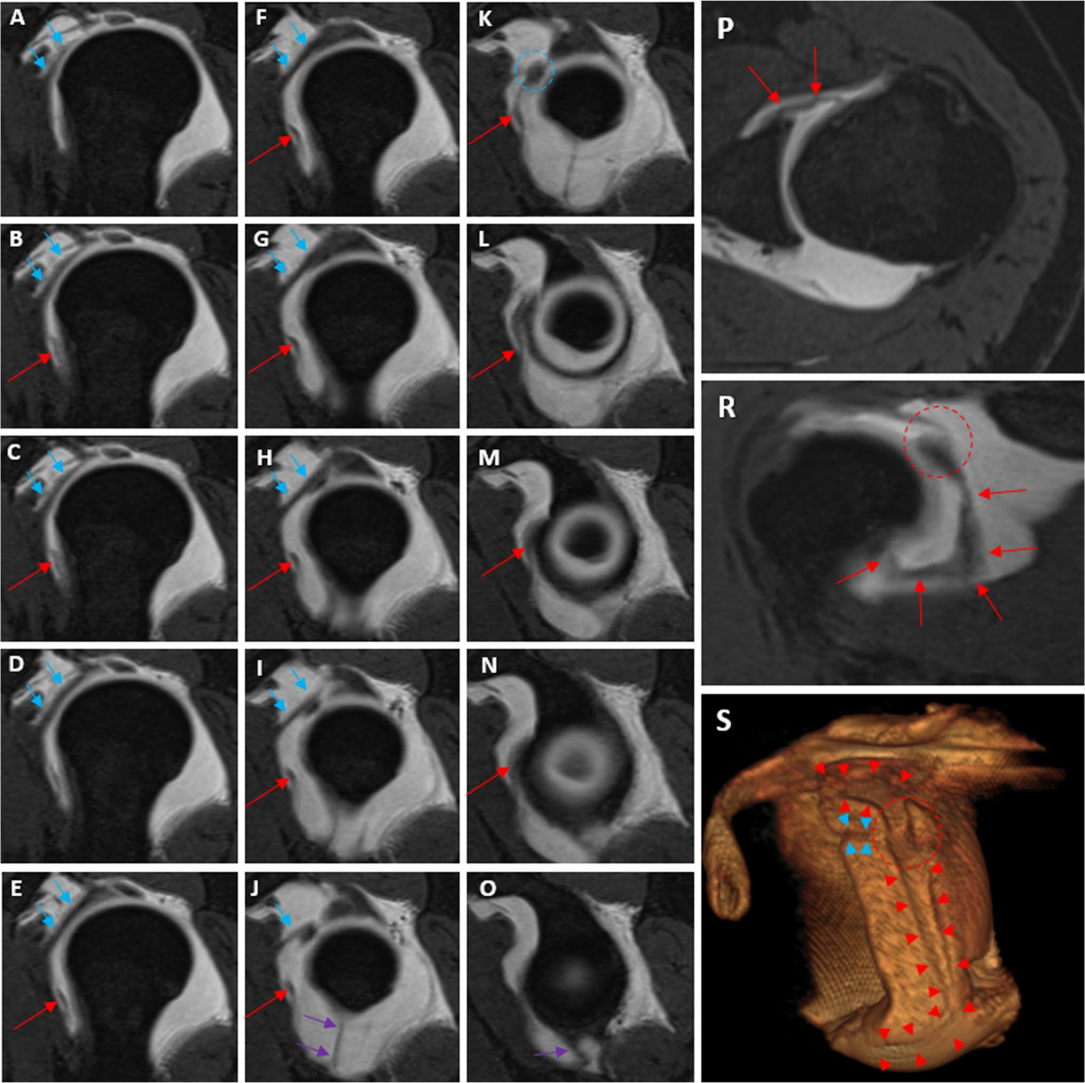
Consecutive sagittal oblique **A**–**O**, axial **P**, coronal oblique **R**, and 3D volumetric rendering **S** VIBE T1-weighted MR arthrograms demonstrate clearly SpiGHL (red arrows and red arrowheads) conjugating (circle in frames **K**, **R**, and **S**) with MGHL and other ligamentous structures such as SGHL (blue arrows and blue arrowheads) and AB-IGHL (purple arrows)

**Fig. 5:**
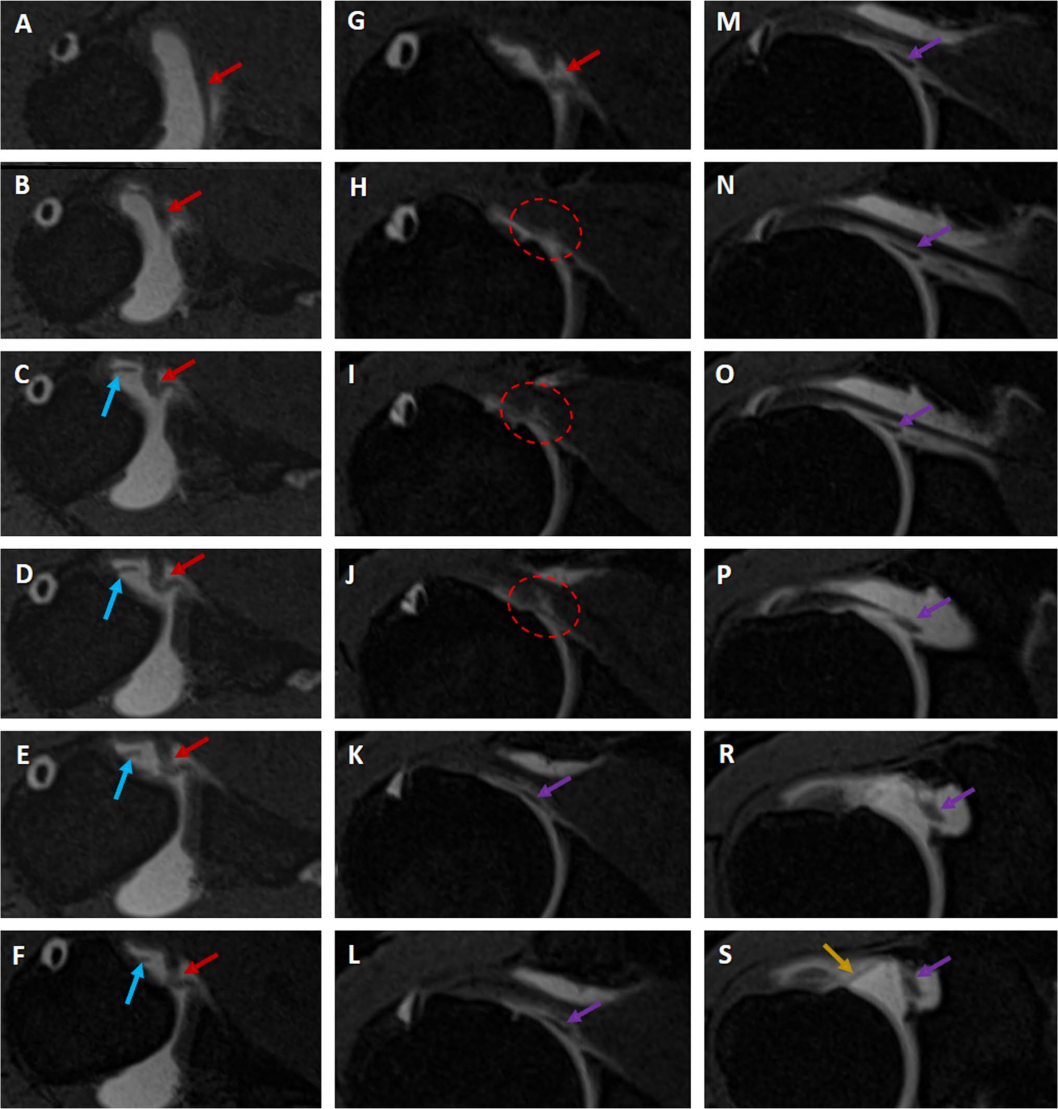
Consecutive axial **A**–**S** VIBE T1-weighted MR arthrograms demonstrate SpiGHL (red arrows) conjugating (circle in frames **H**, **I**, and **J**) with MGHL (purple arrows) and other ligamentous structures such as SGHL (yellow arrow) and AB-IGHL (blue arrows)

The study was conducted between January 2022 and February 2025 and involved 190 patients aged 18–74 years. The demographic data of the patients are presented in Tables 2 and 3. In a cohort of 190 patients, 15 individuals (7.9%) were diagnosed with a SpiGHL. Twenty (10.5%) of 190 patients exhibited high insertion of the AB-IGHL on the anterior glenoid margin. The insertion types of SpiGHL and AB-IGHL on the glenoid rim are presented in Table 3 (Figs. 2 and 3). Among the 15 patients with SpiGHL, 10 (66.6%) demonstrated conjugation of SpiGHL and MGHL (Figs. 4 and 5).

**Table 2:** Table revealing demographic patient data

		Min–max	Ort ± SS
Age		18–74	39.68 ± 13.89
		***n***	**Percentage**
Gender	Male	122	64.2
	Female	68	35.8
Shoulder side	Right	110	57.9
	Left	80	42.1

**Table 3:** Additional demographic patient data

Variable	Category	Spiral ligament (*n* = 15)	High IGHL (*n* = 20)	Spiral ligament conjugating MGHL (*n* = 10)	*p*_1_ (age comparison)	*p*_2_ (gender comparison)
Age (years)	None	18–74 (39.92 ± 14.11)	18–74 (40.44 ± 14.09)	21–45 (34.5 ± 10.76)	0.425	0.955
	Present	21–54 (36.93 ± 10.98)	21–58 (33.30 ± 10.26)	24–51 (34.13 ± 10.45)	0.009	
Gender	Male	112 (91.8%)	109 (89.3%)	6 (75%)	1.000	0.406
	Female	63 (92.6%)	61 (89.7%)	2 (50%)		

*p*_1_ indicates the *p*-value for age comparison between anatomical variation groups and

*p*_2_ indicates the *p*-value for gender distribution comparison between anatomical variation groups

In 160 (84.2%) of 190 MR arthrograms, no additional labroligamentous variation was identified, whereas 13.7% showed a sublabral foramen and 2.1% exhibited a Buford complex. Detailed data on SpiGHL, MGHL, and IGHL are summarized in Table 4.

**Table 4:** Table summarizing MR arthrographic findings of SipGHL, MGHL, and IGHL

		*n*	Percentage
SpiGHL	None	175	92.1
	Present	15	7.9
High IGHL anterior band	None	170	89.5
	Present	20	10.5
MGHL	Aplasia	10	5.3
	Hypoplasia	11	5.8
	Normal	126	66.3
	Double	5	2.6
	Tear	38	20
IGHL	Hypopasia	2	1.1
	Normal	139	73.2
	Multiple	16	8.4
	Tear	13	6.8
	High origin	20	10.5
Insertion types of high IGHL anterior band (*n* = 20)	One o’cklock	2	10
	Two o’cklock	8	40
	Three o’cklock	10	50
Insertion types of SpiGHL (*n* = 15)	Two o’cklock	2	13.3
	Three o’cklock	7	46.7
	Four o’cklock	6	40
SpiGHL conjugating MGHL (*n* = 15)	None	5	33.3
	Present	10	66.7

No statistically significant difference was observed in the average age between individuals with and without SpiGHL (*p* > 0.05). There was no statistically significant difference in the incidence of SpiGHL between males and females (*p* > 0.05). Likewise, the SpiGHL incidence did not differ significantly between the right and left shoulders (*p* > 0.05).

Hypoplasia is characterized as a ligamentous structure exhibiting a thickness of less than 50% in comparison to the contralateral limb or the population average. Out of 190 patients, 11 (5.8%) had MGHL hypoplasia. Among the 11 patients, 5 (45.5%) also exhibited SpiGHL. The incidence of SpiGHL in the hypoplastic MGHL group was significantly higher compared to the normal (5.6%) and tear (5.3%) groups (*p*_1_: 0.001; *p*_2_: 0.004; *p* < 0.05). No statistically significant difference was observed in the occurrence rates of SpiGHL between the normal and tear MGHL groups (*p* > 0.05). Furthermore, in terms of SpiGHL occurrence rates, no statistically significant differences were found among the different IGHL types (normal, multiple, tear, high origin) (*p* > 0.05).

There was no statistically significant difference in the rates of having a high-origin AB-IGHL between individuals with sublabral foramen and those without associated pathology (*p* > 0.05). Similarly, no statistically significant difference was observed between the hypoplastic and normal MGHL groups concerning the rates of having a high-origin AB-IGHL (*p* > 0.05).

There was no statistically significant difference in the mean age between those with and without SpiGHL conjugating MGHL (*p* > 0.05). Additionally, no statistically significant difference was observed in the incidence of SpiGHL conjugating MGHL between males and females (*p* > 0.05). Furthermore, the comparative analysis between right and left shoulders revealed no statistically significant difference in the incidence of SpiGHL conjugating MGHL (*p* > 0.05).

## Discussion

Our study demonstrated a 7.9% frequency of SpiGHL and a 10.5% frequency of AB-IGHL in shoulder MR arthrography, with 66.6% of patients showing SpiGHL in association with MGHL. The prevalence of SpiGHL in existing literature varies considerably. For instance, Merila et al. [6] documented the absence of SpiGHL in an MRI-assessed cadaver yet identified it in all six specimens during both MR arthrography and dissection. In a subsequent study, Merila et al. [7] observed SpiGHL in 100% of cadaver dissections but noted an arthroscopic visibility of merely 44.9%. In contrast, Massimini et al. [9] did not observe SpiGHL in their examination of ten cadavers. These inconsistencies likely arise from methodological differences, including sample selection, imaging techniques, and criteria for ligament identification.

Several factors may explain the relatively low incidence of SpiGHL in our study. First, inclusion of live individuals with shoulder complaints may lead to complicated assessment due to minor shoulder movements. Second, we excluded focal capsular thickenings of < 2 mm from the classification of SpiGHL because of the absence of histological or arthroscopic correlation to avoid overestimation. Third, in this retrospective study, variations in shoulder positioning during imaging—ranging from neutral to external or internal rotation—may have contributed to underrepresentation. Fourth, despite the use of advanced imaging techniques, the absence of gold standard modalities, such as arthroscopy and dissection, limited the precision of our findings. Lastly, differences in expertise between radiologists and anatomists may have influenced the identification of ligaments, as radiologists might be less acquainted with the intricate anatomy of SpiGHL.

The role of SpiGHL in maintaining the biomechanical stability of the shoulder joint is particularly significant. Our findings underscore the consistent presence of SpiGHL in patients with underdeveloped MGHL, suggesting a compensatory mechanism. This adaptive behavior is consistent with the functional synergy observed in MGHL, SpiGHL, and AB-IGHL. The MGHL primarily resists anterior translation and external rotation, especially during mid-range abduction [9]. MGHL hypoplasia is characterized by a ligament thickness that is less than 50% of the anticipated norm, as determined through comparative analysis of axial and sagittal imaging. When the MGHL is hypoplastic, the SpiGHL may take on a more prominent role in resisting external rotation forces, thus contributing to joint stability [10]. Similarly, the AB-IGHL serves as the primary restraint against anterior translation during high degrees of abduction, a critical function for stable shoulder dynamics [11].

The interplay between these ligaments may be influenced by anatomical variations. For instance, the elevated position of the AB-IGHL may significantly alter the stress distribution, highlighting the adaptive importance of SpiGHL within the GHL framework [12]. Advanced arthroscopic techniques have enhanced our understanding of the roles of these stabilizers [13]. Although frequently underrepresented in clinical discussions, the SpiGHL has been increasingly recognized as a distinct anatomical structure originating from the glenoid labrum attached near the lesser tuberosity [14]. Its proximity to MGHL and AB-IGHL suggests a supportive role within the broader capsuloligamentous framework [4].

The increased prominence of SpiGHL in patients with MGHL hypoplasia raises intriguing questions about its developmental and adaptive mechanisms. The prevailing hypothesis suggests that this reflects an adaptive remodeling process to compensate for the MGHL deficit. Such adaptation might involve structural and functional changes that enhance SpiGHL’s ability to resist anterior translation and external rotation. Further biomechanical studies are necessary to comprehensively evaluate SpiGHL’s load-bearing capacity and interactions with MGHL and AB-IGHL under varying physiological conditions.

Numerous MR imaging studies have been conducted to evaluate the anterior labroligamentous structures and even the posterior capsular folds of the shoulder joint [15–18]. The primary objective of our research was to elucidate the imaging characteristics of the SpiGHL and its relationship with other ligaments that have not been adequately defined. In this extensive series, volumetric MR arthrography sequences were presented in addition to conventional MR arthrography images. The MR arthrography sequence we used (3D volumetric interpolated breath-hold examination (VIBE)) allowed for the conversion of images obtained in a single plane into multiplanar and 3D representations with the aid of workstations [19–22]. This approach enabled us to more accurately assess the capsular–intra-articular course and attachment variations of GHLs.

Our study has several limitations. First, given the retrospective nature of this study, our MR arthrography protocol was not optimized for evaluating the SpiGHL. Second, the majority of our patient images were captured in a neutral position. MR arthrograms obtained through positional maneuvers of the shoulder could potentially serve as a more effective method for revealing the SpiGHL and its relationship with other ligaments. Third, conditions like excessive joint distension may adversely affect SpiGHL visualization and its conformings. To enhance diagnostic sensitivity, prospective MRI arthrographic studies focused on this aspect with optimized joint distension could be beneficial. Lastly, the findings of our study were not corroborated by gold standard methods, such as arthroscopy or cadaveric studies.

In conclusion, this study highlights the intricate functional and anatomical relationships among the SpiGHL, MGHL, and AB-IGHL groups, thereby elucidating their collective contribution to shoulder joint stability. By addressing existing gaps in the literature, our findings pave the way for future research aimed at exploring the biomechanical significance and clinical implications of these minor stabilizers.

## Data Availability

The data that support the findings of this study are not openly available due to reasons of sensitivity of human data and are available from the corresponding author upon reasonable request.

## Abbreviations

AB-IGHL: Anterior band of inferior glenohumeral ligament
CT: Computed tomography
GHL: Glenohumeral ligament
MGHL: Middle glenohumeral ligament
MR: Magnetic resonance
SLAP: Superior labrum from anterior to posterior
SpiGHL: Spiral glenohumeral ligament
VIBE: Volumetric interpolated breath-hold examination
